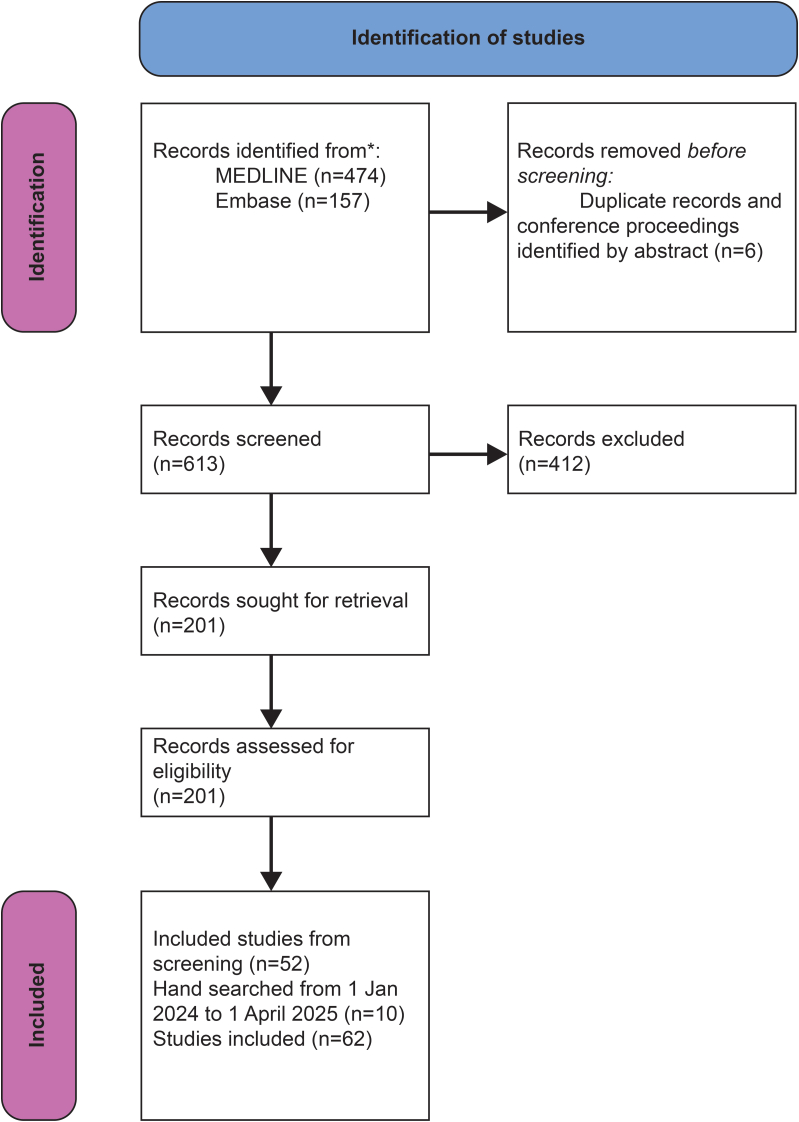# Strategic consensus on the clinical translation of advanced therapies in paediatric rare neurological disorders

**DOI:** 10.1016/j.neurot.2026.e00921

**Published:** 2026-05-12

**Authors:** Natalie Y. Lim, Christian E. Meagher, Ann Bye, Michelle Lorentzos, Russell C. Dale, Shekeeb Mohammad, Katherine B. Howell, Elizabeth E. Palmer, Ian R. Woodcock, Emma Macdonald-Laurs, Anne Preisz, Nadia Badawi, Sandra T. Cooper, Stephen C. Hille, Toby N. Trahair, Anita Cairns, Nicholas Smith, Michelle A. Farrar, Didu S. Kariyawasam

**Affiliations:** a Department of Neurology, Sydney Children's Hospital, Sydney, New South Wales, Australia; b Discipline of Paediatrics and Child Health, School of Clinical Medicine, University of New South Wales, Sydney, New South Wales, Australia; c Sydney Children's Hospitals Network, Sydney, New South Wales, Australia; d School of Medical Sciences, Faculty of Medicine and Health, University of Sydney, Sydney, New South Wales, Australia; e Kids Neuroscience Centre, Kids Research, The Children's Hospital at Westmead, Sydney, New South Wales, Australia; f Department of Neurology, Royal Children's Hospital, Melbourne, Victoria, Australia; g Neuroscience Group, Murdoch Children's Research Institute, Melbourne, Victoria, Australia; h Department of Paediatrics, University of Melbourne, Melbourne, Victoria, Australia; i Rare Diseases NSW, Sydney, New South Wales, Australia; j Centre for Clinical Genetics, Sydney Children's Hospitals Network, Sydney, New South Wales, Australia; k School of Medicine, University of Notre Dame Australia, Sydney, New South Wales, Australia; l Sydney Health Ethics, University of Sydney, Sydney, New South Wales, Australia; m Cerebral Palsy Alliance Research Institute, Specialty of Child and Adolescent Health, Sydney Medical School, The University of Sydney, Sydney, New South Wales, Australia; n Grace Centre for Newborn Intensive Care, The Children's Hospital at Westmead, Sydney, New South Wales, Australia; o The Children's Medical Research Institute, Sydney, New South Wales, Australia; p FOXG1 Research Foundation, Australia; q Kids Cancer Centre, Sydney Children's Hospital Randwick, Sydney, New South Wales, Australia; r Neurosciences Department, Queensland Children's Hospital, South Brisbane, Queensland, Australia; s Department of Neurology and Clinical Neurophysiology, Women's and Children's Health Network, South Australia, Australia; t Discipline of Paediatrics, School of Medicine, University of Adelaide, South Australia, Australia

**Keywords:** Precision medicine, Individualized medicine, Neurologic disorders, Implementation, Translation

## Abstract

Advanced therapies (ATs), including gene and stem cell therapy, hold great potential for preventing and ameliorating many rare neurological disorders (RNDs) in children. These technologies are set to expand across modalities, potentially disrupting and augmenting conventional therapeutic pipelines, with the rapid pace of development highlighting data gaps and implementational challenges. We conducted a two-round modified Delphi study to co-develop a practice framework supporting the safe and effective application of advanced and/or experimental neurotherapeutics for children with rare neurological disorders within a public health ecosystem. The study generated 101 consensus recommendations encompassing criteria to 1) facilitate equitable and timely therapeutic access, 2) optimise transparent communication and shared decision making with families, 3) incorporate disease and patient level considerations for minimising risk and optimising safety within advanced therapeutic research, 4) strengthen resourcing of health systems to enable longitudinal evaluation of treatment effects and safety. Embedding this framework into practice will depend on enhancement of workforce training, establishment of digital infrastructure, fit-for-purpose clinical environments and education and engagement of patients, families and the broader community.

## Introduction

Paediatric rare neurological disorders (RNDs), defined as conditions affecting less than 1 in 2000 people, collectively represent a substantial global disease burden [1,2]. While significant progress has been made in developing effective therapies for these conditions, fewer than 5% have approved targeted treatments [3,4]. However, the introduction and advancement of innovative genomic technologies, has fuelled the expansion of a therapeutic armoury of advanced therapies and experimental neurotherapeutics for children with RNDs (Box 1). As such, there are currently >600 neurotherapeutics advancing rapidly through the research and development pipeline [5].

Box 1 **Key definitions used within the recommendations**

*Advanced therapies* are medicines for human use that are based on genes, tissues or cells. These can include cell or gene therapies.

*Neurotherapeutics* encompass medications, devices and procedures that can be used for the treatment or management of neurological conditions.

*Experimental neurotherapeutics* are i) treatments that are translated from novel scientific discoveries and development, and/or ii) treatments that are an extension of therapy beyond the standard duration or using a conventional treatment in a different context (i.e. repurposed drugs).

The experimental neurotherapeutics considered within these recommendations focus on new advances in neurosciences that offer therapeutic potential, for example advanced therapies, personalised antisense oligonucleotide (ASO) therapies, ribonucleic acid (RNA) therapies, immunotherapies, neurostimulation and laser therapy for epilepsy.

Alt-text: Box 1

Whilst accelerated access to treatments has been driven by international policy directives and undoubtedly benefited consumers, there is a significant bottle neck when it comes to clinical translation of advanced therapeutics [6]. The imperative to anticipate and build healthcare readiness for emerging therapeutics is exemplified by spinal muscular atrophy (SMA) with the advent of three advanced therapeutics coupled with newborn screening [[7], [8], [9]]. Learnings from the implementation of these therapies include a paucity of knowledge on how to best stratify children to emerging first and second-generation treatments, concurrently provide efficient yet equitable access to children within a narrow therapeutic window, adapt current health system infrastructure for delivery and monitoring of treatments and capture real world outcomes that inform therapeutic decision beyond the data generated through clinical trials [9,10]. Whilst SMA is an exemplar condition, the rapidly changing frontier of advanced therapeutics means that such obstacles and barriers will be amplified across conditions, barriers including but not limited to high treatment cost and reimbursement challenges, limited availability of specialised treatment centres and infrastructure and uncertainties surrounding long-term efficacy and safety monitoring.

Advances in biotechnologies, including base editing, antisense oligonucleotide therapy and adeno associated viral vector mediated gene therapy, provide a template to build approaches for highly personalised therapies aimed at treating individuals where the causative pathogenic variant is unique or shared by only a few persons (referred to as ‘n = 1’ therapies) [[11], [12], [13]]. The platform nature of such technologies has already resulted in Australian clinicians and health services receiving requests for their potential clinical use for individual children. Consequently, guiding principles are needed to responsibly streamline research, innovation and broader translation of these therapies. In developing such principles, it is essential to consider which patients are most appropriate for treatment, how interventions should be optimally delivered, risks minimised, and benefits captured in a systematic way to facilitate broader access.

### Aim

The purpose of this study was to co-develop consensus based best practice recommendations for the responsible clinical use of advanced and/or experimental neurotherapeutics for children with RNDs within the Australian public health system.

## Materials and methods

### Steering committee

A steering committee (NL, MF, DSK and CM) was formed and initially discussed the scope and aims of the project, formulating eight key topics for consideration (Supplementary Table 1). A panel of nineteen participants were purposively recruited from all states and territories of Australia and encompassed experts in paediatric medicine, clinician researchers, clinical trial experts, scientists, ethics, research governance, development of experimental neurotherapeutics and lived experience and advocacy (Table 1).

**Table 1:** Characteristics of Delphi panel participants.

	Round 1	Round 2
Primary role		
Paediatric neurologist	11	9
Paediatric oncologist	1	
Neonatologist	1	1
Scientist	1	1
Parent/Advocacy group leader	1	1
Ethics	2	1
Governance	1	1
Genetics		1
State of residence		
New South Wales	12	11
Victoria	3	3
South Australia	1	
Western Australia	1	1
Queensland	1	
Years of experience		
0–9 years	2	2
10–19 years	9	8
20–29 years	4	2
30+ years	3	3
Gender		
M	6	4
F	12	11

### Supporting literature review

A scoping literature review was conducted to identify evidence related to the ethics, experience or implementation of advanced and experimental neurotherapeutics in paediatric RNDs. The search was conducted using two databases, Embase (Ovid) and PubMed from January 1, 2014 to January 1, 2024. Keywords (including synonyms of) “molecular targeted therapies”, “genetic therapies”, “advanced therapies”, “experimental therapies”, “experimental neurotherapeutics” or “precision medicine “were combined with “neurology” and “nervous system diseases” and “rare disease” and “neurogenetic”. The search included publications in the English language and was limited to the paediatric population (up to 18 years of age). Ancestry searching and citation chaining were performed to identify additional literature. Reports of targeted therapies in neuro-oncology were excluded as the translational context differs sufficiently regarding therapeutic intent, clinical trial endpoints, funding structures and regulatory models. Studies were selected based on relevance and significance to addressing the key issues in the translation and implementation of advanced therapies. A narrative synthesis was carried out and organised to characterize clinical practices, processes, gaps and limitations.

### Consensus process for recommendation development

The aim, scope, and narrative synthesis from the literature review were presented to the panel through an online workshop. Here, the panel provided feedback on the population, settings and clinical priorities to be considered in the recommendations. Following the workshop the steering group collaborated and iteratively developed key priorities for consensus, grouped into four domains: (1) access pathways and responsibilities for trials and treatment (2) communication and engagement with families (3) trial and treatment eligibility and patient selection (4) evaluation of the effects of trials or treatment and implementation of treatment plan (Supplementary Table 2).

A modified Delphi methodology was used, with up to three survey rounds planned. Survey items, with supporting notes and examples, were presented to panel members in an electronic survey using REDCap (hosted at University of New South Wales Sydney) on July 8th^,^ 2024 and November 14th^,^ 2024. Panel members responded to the survey items in their area(s) of expertise/scope of practice. Responses each statement were measured using a 5-point Likert scale (strongly agree/agree/neither agree nor disagree/disagree/strongly disagree). In addition, optional comments for each item were collated as written text as appropriate. Consensus was defined *a priori* as achieving agreement/strong agreement among at least 75% of participants.

The first survey (Q1) was analysed using descriptive statistics and qualitative content analysis. Findings were summarised and reviewed by panel members in a second online workshop with modification of items categorised as achieving near consensus (‘neither agree or disagree’) or no consensus (‘disagree’). The second survey (Q2) was developed by the steering committee, incorporating items from the first round of the Delphi process that had achieved near or no consensus. A feasibility rating was included in Q2 where panel members were asked to consider implementation cost, scalability, resource needs and stakeholder's time. This was measured using a 5-point Likert scale (very feasible/feasible/neutral/not feasible/very not feasible) and feasibility scores were grouped according to domain.

## Results

### Scoping review

The literature search yielded 631 articles, with 52 publications included in the scoping review (Supplementary material figure 1).

Eleven articles discussed clinician and patient experiences and challenges following the implementation of SMA advanced therapies as standard of care (Supplementary Material 1.3). Common topics were the emergence of a neurogenetic emergency, new disease trajectories and changing models of care and the need for standardised outcome measures to harmonise the reporting of real word data.

Beyond SMA, an expanding therapeutic pipeline across paediatric epilepsies, movement disorders, neuroimmune and neurodevelopmental conditions was evident, reflecting the landscape in which rational drug repurposing, targeted molecular therapies, gene replacement and neuromodulation were under active investigation or entering clinical use (Supplementary material 1.3). However, the ecosystem appeared to remain fragmented across clinical, scientific, regulatory and social dimensions. Although a few consensus guidelines and frameworks were noted within the evidence based they were often disease-specific or geographically limited [[14], [15], [16], [17]]. The literature collectively highlighted an ongoing and urgent need for coordinated health system readiness including robust infrastructure, standardised operational frameworks and streamlined regulatory pathways to support equitable access, consistent monitoring and long-term evaluation [14,15,18]. Furthermore, the literature highlighted a critical demand for scalable education and communication strategies to enhance clinician expertise and patient understanding [19,20]. Co-developed standards for the responsible and optimised clinical use of innovative therapies for children with RNDs were lacking, yet critical to drive progress and enable consistent, transparent, equitable and safe clinical research practices. These insights directly informed the design of our survey, helping to scaffold key thematic issues such as stakeholder and health system readiness, perceived barriers to access, ethical considerations and the role of communication and shared decision making with families, in advanced therapeutic uptake.

### Delphi survey

There were 19 panellists, with representation from 5/6 Australian States. The first Delphi round included 108 key statements, created based on the clinical practice of the committee and available literature.

Q1 was completed by 18/19 (94.7%) participants with 97/108 (89.8%) items meeting consensus (Supplementary Table 3). In the second workshop (Q2), 11 items were brought forward for consideration by 15/19 (79%) participants. Here 3/11 (27%) recommendations that did not meet consensus were considered for amendment as the primary issue was the language used. Statements regarding medical treatment overseas did not meet consensus and therefore all statements pertaining to this were removed. Two new recommendations were generated following the second workshop (Supplementary Table 4).

**Fig. 1:**
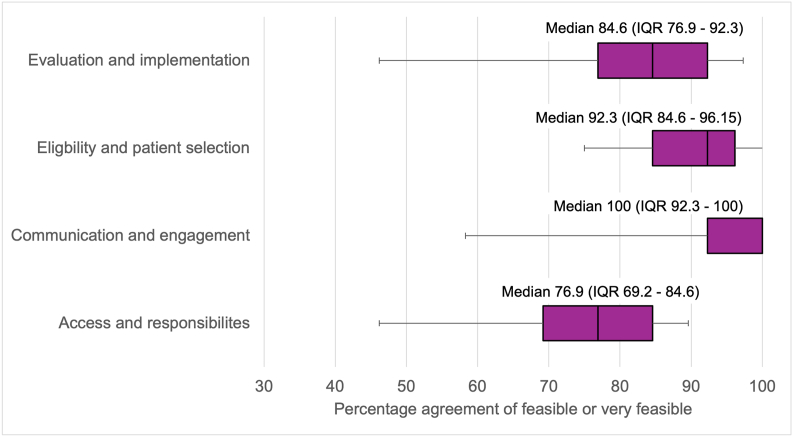
Feasibility scores for consensus recommendations for advanced therapies in paediatric rare neurological disorders.

Overall, the Delphi process identified 101 recommendations for the clinical implementation of advanced therapies in paediatric neurology, structured across four key domains. Feasibility scores were high across all domains with median scores values between 76.9 and 100 (Fig. 1).

First, participants highlighted the need for a coherent and transparent information ecosystem to support both clinicians and families. As these therapies increasingly emerge outside traditional clinical pathways, clear guidance on access, institutional responsibilities and care pathways is essential to ensure equity.

Illustrative example: Nusinersen, an intrathecally administered antisense oligonucleotide, received FDA approval in 2016 whilst at the same time, an expanded access program for patients with SMA type 1 (the severest and infantile onset form of the condition) was initiated in Australia alongside an expedited regulatory review [10]. When the therapy became available through the Pharmaceutical Benefits Scheme (PBS) in 2018 significant coordination was required with parents actively reaching out to clinicians to organise lumbar punctures and initiate treatment. However, equitable access remained challenging particularly in regional and rural areas due to difficulties in service delivery. Communicating the risks and benefits of the treatment to culturally and linguistically diverse communities also proved complex. This period marked a turning point, reframing SMA as a neurogenetic emergency and contributing to the adoption of SMA newborn screening in Australia..

Box 2 **Domain 1: Access pathways and responsibilities for trials and treatment. Consensus was achieved for 22 of 28 statements evaluated; these 22 statements form the basis of the recommendations in Domain 1.**

**Collaboration**.

A collaborative paediatric clinical trials alliance by which the experience and expertise of those studying and utilising advanced and experimental neurotherapeutics can be shared efficiently and transparently should be a priority in local and national policy.

**Peer review**.

A peer review advisory group involving clinicians with expertise in advanced and experimental neurotherapeutics would be valuable as an adjunct to established organisational pathways for clinicians to evaluate best practice, support decision making and share knowledge and experience.

**Evaluation of health system readiness**.

Decisions to access advanced therapies and experimental neurotherapeutics should follow established organisation pathways considering.

• The ability of the health system to accept responsibility within a local policy framework.
• The capacity of the clinical and health service to implement the proposed treatment and post-treatment activities.
• Appropriate resources, expertise and authorisation and approvals pathways

**Dissemination of treatment opportunities**.

Health services should formulate and implement a dissemination plan so that treatment opportunities are shared across the population.

• Where there is competing space on a clinical trial or managed access program and after review of capacity and anticipated clinical benefit, the process of allocation to receive treatment should be transparent and communicated to families.
• Health services should collaborate with community services and patient advocacy groups to facilitate dissemination of treatment opportunities to children and families with neurological conditions.
• The dissemination plan should have specific strategies to target underrepresented groups, including but not limited to families with low health literacy, socioeconomic disadvantage, culturally and linguistically diverse populations, Aboriginal and Torres Strait Islander and rural/regional children and their families.

**Parent/Caregiver Involvement**.

When parents/caregivers are involved in the funding and/or development of therapies, evaluation of the following is recommended.

• Potential conflicts of interest including partnerships with not-for-profit organisations and philanthropic foundations.
• Formulation of a plan to incorporate equitable access for other suitable patients and families.
• Privacy risks in the case of crowdfunding or compelling cases receiving media attention.

**Infrastructure and resourcing**.

To allow the best opportunities for the clinical development and implementation of new therapies, the following should ideally occur.

• Ongoing education and professional development of all stakeholders.
• A local ethics and governance officer specific to advanced therapies.
• Community engagement to understand families' and children's information needs and to communicate effectively, supporting informed decisions.
• Consumer partnerships to facilitate the co-design health care to meet their needs.
• Standards for clinicians and centres.
• Promotion of multidisciplinary teams and research integrated clinical care models.

Alt-text: Box 2

Secondly the importance of sustained, transparent communication with families was emphasised particularly in relation to managing expectations and defining decision-making boundaries.

Illustrative example: Neuromodulation therapies, such as deep brain stimulation (DBS) and intrathecal baclofen (ITB), offer important options for managing drug-resistant epilepsy and movement disorders. While these interventions can reduce symptoms and may allow for decreased medication use, they are not curative. Due to their invasiveness and variable outcomes, establishing clear start and stop criteria alongside transparent, ongoing communication with families is essential. Educational materials are vital but frequently lack comprehensive patient journey insights, fall below readability benchmarks, and rarely incorporate multimedia or patient perspectives. While firsthand accounts can enhance engagement, they may skew perceptions by underplaying risks and overstating benefits, risking unrealistic expectations [21]..

Box 3 **Domain 2: Communication and engagement with families. Consensus was achieved for 36 of 38 statements evaluated; these 36 statements form the basis of the recommendations in Domain 2.**

**Clinician communication**.

Once an advanced therapy or experimental neurotherapeutic is identified and considered for administration, clinicians should ensure that communication with the child and family/caregiver includes the following components and that these are clearly documented:

Language and cultural support.

• Families whose primary language is not English must be supported by a qualified professional interpreter
• Families identifying as Aboriginal or Torres Strait Islander should be offered support from an Aboriginal Health Liaison Officer

Access to Support Resources.

• Offer connections with appropriate psychological, medical and/or social work support
• Referral to relevant patient advocacy groups should be offered to support decision-making
• Where available, families should be provided with high-quality, evidence-based written or multimedia materials

Shared understanding of the therapeutic process.

• Discuss the stages nature of decisions regarding initiation, continuation and discontinuation
• Describe the full access process, including any wait period
• Establish expectations for the mode, frequency and responsiveness of communication between the clinical team and family
  Time sensitive situations
• In medical emergencies where delays in treatment can adversely change future outcomes for the child, families/caregivers should be immediately informed of the benefit of early treatment to aid their decision making.

**Establishing start and stop criteria**.

Start and stop criteria should be discussed and established with the child and family/caregivers and documented, ideally early in communications, and before treatment initiation to set expectations.

Start criteria discussions should address.

• Defined treatment objectives
• Planned methods, frequency and duration of outcome evaluation
• Evaluation of safety signals
• Rationale for treatment that includes scientific justification and plausible efficacy

Stop criteria discussions should address.

• Treatment objectives not met from the perspective of the child, family or managing clinician
• Severe adverse drug reaction or cumulative side effects necessitating cessation

**Informed consent requirements**.

Informed consent must include transparency around the following.

• Anticipated outcomes, including explicit explanation that the therapy may be non-efficacious.
• The potential to preclude the child in accessing future clinical trials or treatments.
• The potential short and long-term side effects and uncertainties of the treatment itself.
• The potential side effects or risks of the administration/delivery process.
• The variability in sustained access to experimental neurotherapeutics over the longer term, even if deemed beneficial (relevant within the clinical trial domain).

**Ongoing** **support and oversightand oversight**.

The most appropriate healthcare professional(s) for contact regarding experimental trials and treatments should be agreed upon by all and contact details provided.

A second clinician or researcher with technical knowledge and understanding of the ethical applications of advanced therapeutics should be offered if there is uncertainty.

Alt-text: Box 3

Ethically robust approaches are essential, grounded in transparent assessments of potential benefit. Achieving this requires improved predictive tools and adaptive decision-making frameworks that evolve alongside the emerging evidence base.

Illustrative example:

The use of autologous ex vivo hematopoietic stem cell gene therapy (HSCT-GT) for presymptomatic and early symptomatic metachromatic leukodystrophy (MLD) represents a pivotal developmental in the management of this rare disease. Historically, treatment options were limited to enzyme replacement therapy and allogenic hematopoietic cell transplantation. Recent evaluations of published positive outcomes for HSCT-GT in advanced-stage MLD have highlighted concerns regarding the absence of clearly defined inclusion criteria for ‘advanced disease status’ and a lack of comprehensive data to adequately assess the safety and efficacy of this intervention in this group [22]. While it is recognized that cases on the borderline pose challenges in determining potential therapeutic benefit, a critical appraisal of the available evidence remains essential. An MLD eligibility treatment panel was considered a valuable tool in navigating these complex decisions [23]..

Box 4 **Domain 3: Trial and treatment eligibility and patient selection. Consensus was achieved for 22 of 23 statements evaluated; these 23 statements form the basis of the recommendations in Domain 3.**

**Inclusion criteria (outside of clinical trials)**

All of the following should be addressed.

• Defined and documented expectations about possible benefits, harms and uncertainties
• The child and family can continue to receive multi-disciplinary care
• Other safe and effective disease modifying treatments are limited or unavailable
• The potential for benefit as informed by:
  o Patient level characteristics – comorbidities, child and family preferences, ability to adhere to post treatment surveillance and follow up
  o Disease level characteristics – stage of disease, reversibility of end organ damage, extent of end organ damage
  o Scientific justification – evidence in similar disease groups, preclinical evidence of benefit

**Advanced neurodegenerative disease considerations**.

In children with advanced-stage neurodegenerative disease, the decision to offer treatment should take into account the potential for.

• Symptom stability
• Maintenance of quality of life and adaptive function
• Patient or proxy beliefs regarding meaningful treatment outcomes
• Achievement of conventional endpoints including:
  o Gains in function.
  o Developmental progression.
  o Reduction in mortality and comorbidities.

**Exclusion criteria (outside of clinical trials)**

Any of the following may justify exclusion.

• Availability of alternative safe and effective disease-modifying treatment
• Treatment cannot be safely administered
• Therapeutic burden is too high in the opinion of the parents and/or the treating team

Alt-text: Box 4

Finally long-term integration of these therapies depends on investment in infrastructure to support outcome monitoring, care coordination and continuous evaluation. Without such systems, even highly promising therapies risk inconsistent delivery and limited real-world impact.

Illustrative example:

Selecting appropriate outcome measures for neurodevelopmental disorders presents unique challenges due to heterogenous presentations and variable developmental trajectories. Recent studies in Rett syndrome illustrate how condition-specific assessments can detect meaningful change. This is exemplified by the use of the Rett Specific Behavioural Questionnaire (RSBQ) alongside the Clinical Global Impression – Improvement (CGI-I) in the trofinetide trials [24,25]. Condition specific tools are most effective when used alongside objective, regulatory grade endpoints such as performance measures or surrogate biomarkers. Together these measures provide a multidimensional view of treatment benefit that aligns with regulatory expectations and supports pathways for reimbursement. Importantly, caregiver and clinician input is critical in defining what constitutes meaningful improvement, ensuring outcomes are both scientifically valid and grounded in real world experience [26]..

Box 5 **Domain 4: Evaluating the effects of trials or treatment and implementing a treatment plan. Consensus was achieved for 21 of 21 statements evaluated; these 21 statements form the basis of the recommendations in Domain 4.**

**Outcome measures**.

Outcome measures should include.

• Conventional clinical endpoints
• A range of meaningful child and family endpoints
• Subjective experiences (such as quality-of-life scales and global impression scores)
• Appropriate relevance to disease stage, function and trajectory

Early engagement with consumers occurs to facilitate co-design of clinical trials and co-development of appropriate endpoints.

Potential surrogates (biomarkers) of disease activity and therapeutic effect should be assessed and incorporated into ongoing research.

**Safety measures**.

An independent review of data and safety assessments and reports should occur.

Safety assessments should include physical exams and targeted assessments related to known class effects.

**Platforms to be used across different conditions and therapies**.

To allow the best opportunities for clinical trial readiness and real-world evaluation of effects, the following should be fostered.

• Have simplified protocols in place to conduct evaluations prior to administration of treatments E.g., parallel collection of natural history, run in data, use of retrospective data.
• Have common protocols in place to collect data following initiation of treatment, and potentially long-term.
• Have measures for rapid data sharing to accelerate learning.

Alt-text: Box 5

## Discussion

The concept of precision and personalised medicine in RNDs is gaining momentum, fuelled by using tailored (advanced) therapeutic strategies that align with a child's own set of unique biopsychosocial, to magnify health outcomes. Coupled with this, the application of diagnostic and genetic analysis tools has further increased the number of identified eligible children, who could have recourse to advance therapeutics as part of their therapeutic armoury, now and in the future [27]. However, the precision medicine paradigm is considered a potentially disruptive innovation, that challenges conventional and existing standards of care and practice, redefining how we characterize our patients and select safe and effective treatments within an evidence base that is not necessarily informed by clinical trials but an expedited and more patient-specific drug development pipeline [28].

Aligning with the core pillars of national rare disease policy [29], this study addresses emerging implementational barriers to provide a practical framework that enables children to have fair and transparent access to research opportunities, whilst empowering communication between decision makers to explore the risks, benefits and uncertainties of treatment. Further, this study embeds the imperative for research as a core business of clinical care, to facilitate patient selection and stratification whilst developing methods to monitor therapeutic response and safety signals in advanced therapeutic. The current study's results attest to an important strategy change in RND healthcare to a ‘top down’ approach in which decision makers within health systems drive forward the enablers for precision medicine, ensuring that current ways of working are reconfigured to shape sustainable innovations in healthcare [30,31].

Due to the desired rapid evolution and exchange of knowledge and information at the intersection between research and clinical care, relevant and specific ethical questions arise by bringing advanced therapeutics to clinical care. A peer consultation process and advice on approaches and review amongst clinicians as determined in this study appears to be the foundation step that enables advanced therapeutics to be effectively reviewed and compared against a patient's unique biological and psychosocial signature. This replicates prior work that emphasises interdisciplinary collaboration and the involvement of all relevant stakeholders to assess the rationale, risks and benefits of proposed treatments [30,32]. Setting individualized therapeutic expectations with parents early on and establishing checkpoints for ‘go/no’ access and continuance of treatment as seen in our study findings, remain a fundamental aspect of ensuring that the child's needs remains centre most in the often changing risk-benefit equation [14,33].

Communication and engagement with the RND community was one of the core themes within this study. Expert consensus acknowledged the changing role of consumers and advocates in rare disease research, addressing the need to manage conflicts of interest for families funding or collaborating with sponsors for their own treatments, setting therapeutic expectations early within the decision-making process and ensuring transparency within the RND community on patient selection processes for competitive recruitment. Families with children who have RND's acknowledge a high-risk appetite for research and advanced therapeutics [34], however, trust and engagement in the healthcare system can only be preserved when families share in the decision-making process, with information provision that allows for a full understanding of the dynamic risk-benefit profile and uncertainties of advanced therapeutics [35,36]. This is particularly pertinent in a space where access to a treatment may preclude options for future (second generation) interventions [14]. The need for psychological and advocacy support was a finding of this study and has been defined as a key element to enable health system readiness for advanced therapeutics [14,[37], [38], [39]].

The evaluation of effects of treatment requires specific consideration in advanced therapeutics within the RND landscape, as addressed by our study findings. This aligns with prior studies that emphasise the need to capture disease manifestations that are both acceptable to the expert disease clinical community and meaningful to consumers through patient and parent reported outcomes, emphasising selection or development of outcome measures should involve the RND community [11,40]. In RNDs treatment effects often unfold gradually over long periods. Unlike diseases with more binary immediate benchmarks of survival or cure, improvements in cognition, development and function are complex, influenced by age, environment, long-term functional gains and supportive care. This complexity requires sensitive, age-appropriate outcome measures, extended follow up to fully capture the impact of interventions over time and consensus clinical guidelines. To support this, robust infrastructure and strategic frameworks are essential, with rare disease care centres emerging globally as effective models for integrating scientific research and clinical care [19,41]. Here, there remains a scientific and ethical imperative to collate, document and analyse outcomes so that learnings can be shared and applied at larger scale, particularly when considering the emergence of platform technologies within the drug development pipeline. The linking of data to centralised pan-national or international data registries are being considered as a way of standardising core data collection and ensuring data quality for rare neurological conditions across clinical research hubs. These have-to-have flexibility to inform multiple purposes and thus serve the needs of different stakeholders including consumers, clinicians, researchers, payers and industry sponsors, to further inform the evidence base for advanced therapeutic development.

The strengths of this study are its modified Delphi methodology that allows for iterative discussion and systematic feedback from experts who are currently and actively working in the field, informing a domain where there is paucity of high quality and systematic evidence. The limitations in using this methodology include the possibility for inherent bias given anonymity was not maintained throughout the rounds. Dominance and group conformity were considered and moderated with structured facilitation during workshops and the option to provide open text commentary within the surveys.

The recommendations provided here align with both regional policies and frameworks as well as overarching global mandates such as the World Health Organisation Intersectoral Global Action Plan on epilepsy and other neurological disorders [42]. As such, the recommendations reflect a shared focus on sustainable funding, institutional capacity building and active patient and caregiver engagement. An emerging fiscal challenge, both within the Australian context and internationally, is funding high cost advanced therapies. Inequities in access to transformative neurotherapeutics is compounded in low and middle-income countries, who are often excluded from research opportunities due to lack of health system resourcing and capacity. Bridging these access gaps will depend on a paradigm change from traditional cost accounting towards a model that recognises the preservation and optimisation of brain health as a primary asset for national and economic stability [43].

**Fig. 2:**
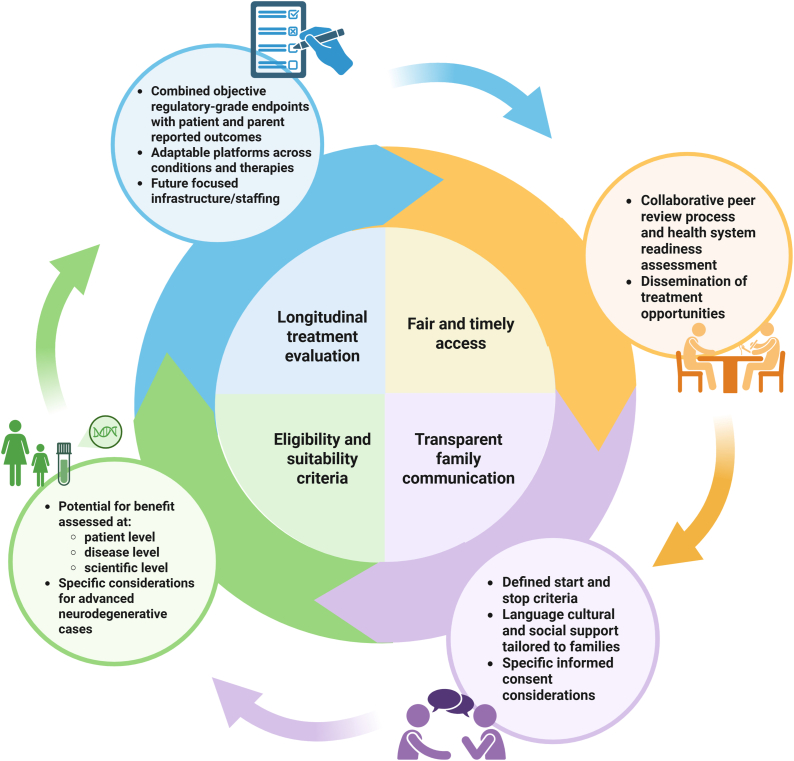
Model of the key domains of advanced therapy delivery in paediatric neurology and their iterative interactions within the health system.

The recommendations are deliberately not prescriptive but rather provide the genesis of an approach for clinicians and health systems that can be adapted to evolving clinical needs and organisation contexts (Fig. 2). The next phase will involve real-world testing which involves applying the recommendations across selected topics and clinical scenarios to identify practical gaps, gather feedback and support iterative refinement. Looking to the future, the long-term integration of this framework into clinical care will hinge on coordinated efforts to establish sustainable funding models, scalable professional development programs, sufficient workforce capacity, and robust systemic infrastructure [44]. Effective implementation is unlikely to succeed without reliable financing mechanisms that extend beyond one off time limited research grants. Similarly, clinician readiness will depend on availability of flexible training models that address foundational knowledge and evolving competencies in advanced therapeutic approaches. The increasing expectation that clinicians keep pace with emerging treatments, often requiring significant time to evaluate emerging evidence, coordinate care and engage with external stakeholders, must be formally recognized and compensated. These activities typically fall outside funded research roles and current models of clinical workload. Meeting these escalating demands necessitates the expansion of the clinical workforce through creation of additional positions, ensuring adequate numbers of skilled professionals can deliver and oversee these therapies with the requisite expertise. In parallel, the development of digital infrastructure and data governance systems that support secure information exchange are critical in facilitating collaboration across sectors and facilitates timely and efficient evaluation.

Advanced therapies offer the potential to improve health outcomes for children with RNDs. This study generates practical guidance, positioning the implementation of advanced therapeutics in RNDs as the next frontier of a proactive precision medicine paradigm. This co-developed guideline, provides a practical, clinician-informed framework for sustainable care that is fit for purpose, reflects consumer priorities, informs policy development and is responsive to evolving approaches in rare disease research and clinical translation.

## Author contributions

NL, DK, MF, and CM conceived the concept for this consensus initiative and comprised the steering group. They also led the writing group and drafted the manuscript. All other authors served as panel members, contributing to the review of evidence, providing expert input during the consensus process, and critically reviewing the manuscript. All authors approved the final version for submission.

## Declaration of competing interest

The authors declare that they have no known competing financial interests or personal relationships that could have appeared to influence the work reported in this paper.

## Figures and Tables

**Fig. S1:**