# Supplementary material for: Strategic consensus on the clinical translation of advanced therapies in paediatric rare neurological disorders
Source: Neurotherapeutics. 2026 May 12;23(3):e00921. doi: 10.1016/j.neurot.2026.e00921 (PMC13195611; doi:10.1016/j.neurot.2026.e00921)
Supplement: Multimedia component 1 [file mmc1.docx]

**Strategic consensus on the clinical translation of advanced therapies in paediatric rare neurological disorders – supplementary material**

Table of Contents

[Section 1 – Review foundation and topic selection 2](#_Toc227826207)

[1.1- Initial scope and purpose 2](#_Toc227826208)

[1.2 – Expert panel selection 3](#_Toc227826209)

[1.3 - Scoping review 4](#_Toc227826210)

[1.4 – Topic generation 7](#_Toc227826211)

[Section 2 – Workshops and Questionnaires 10](#_Toc227826212)

[2.1 - Workshop 1: Expert panel consultation and thematic development 10](#_Toc227826213)

[2.2 - Questionnaire 1 13](#_Toc227826214)

[2.3 - Workshop 2: Review of non-consensus items 20](#_Toc227826215)

[2.4 - Questionnaire 2 23](#_Toc227826216)

# Section 1 – Review foundation and topic selection

## 1.1- Initial scope and purpose

**Date of initial scope and purpose document: 16^th^ July 2023**

The purpose of these consensus-based recommendations is intended to assist individuals in the application of advanced therapies and/or experimental neurotherapeutics for children with neurological disorders within the Australian public health system. Our findings aim to complement existing organisational and governance pathways with a focus on addressing anticipated clinical challenges in the consideration and implementation of these therapies.

**Population**

The consensus-based recommendations pertain to children under the age of 18 years, diagnosed with neurological conditions and their parents/caregivers seeking to access advanced therapies and/or experimental neurotherapeutics in Australia.

**End users**

The targeted primary end users of these consensus-based recommendations include healthcare professionals involved in the management of children with neurological conditions. Targeted secondary end users include health system planners, managers, and administrators whose organisations provide services for children with neurological conditions and their parents/caregivers.

## 1.2 – Expert panel selection

Expert panel members were identified and recruited by the steering group (NL, MF, DSK, CM) based on those individuals who met the following inclusion criteria (a) paediatric neurologists, physicians, scientists, ethics and governance members, advocacy group leaders or clinical nurse specialists with experience in advanced therapy implementation or research; (b) a record of research output or policymaking in paediatric rare neurological diseases or advanced therapy implementation; and (c) the availability to actively participate in the recommendations development process. To reflect the importance of caregiver perspectives and align with community priorities in the development of these recommendations, the leader of a rare neurogenetic disorder advocacy group, who is also a parent with lived experience, was included on the panel and participated in the same capacity at all workshops and in the questionnaires. In total, the expert panel comprised 19 members recruited via email invitation.

## 1.3 - Scoping review

Two data bases, Embase (Ovid) and PubMed were searched by NL. Searches were conducted from 1^st^ January 2014 to 1^st^ of January 2024. The literature search was updated on 1^st^ of April 2025 to update the panel of any emerging evidence.

**Supplementary Figure 1.** Scoping review study selection

**Identification of studies**

Records removed *before screening*:

Duplicate records and conference proceedings identified by abstract (n = 6)

Records identified from*:

MEDLINE (n=474)

Embase (n=157)

**Identification**

Records screened

(n=613)

Records excluded

(n = 412)

**Screening**

Reports sought for retrieval

(n = 201)

Reports assessed for eligibility

(n = 201)

Included studies from screening

(n = 52)

Hand searched from 1 Jan 2024 to 1 April 2025 (n=10)

Studies included (n=62)

**Included**

Selected references on ATs in SMA and other paediatric neurological conditions provided prior to the first online workshop.

*Eleven SMA articles highlighting clinician and patient experiences and challenges following the implementation of SMA advanced therapies as standard of care.*

1. Aguti S, Malerba A, Zhou H. The progress of AAV-mediated gene therapy in neuromuscular disorders. Expert Opin Biol Ther 2018; 18: 681-693.
2. Balaji L, Farrar MA, D'Silva AM, et al. Decision-making and challenges within the evolving treatment algorithm in spinal muscular atrophy: a clinical perspective. Expert Rev Neurother 2023; 23: 571-586.
3. Butterfield RJ. Spinal muscular atrophy treatments, newborn screening, and the creation of a neurogenetics urgency. Semin Pediatr Neurol 2021; 38: 100899.
4. Farrar MA, Park SB, Vucic S, et al. Emerging therapies and challenges in spinal muscular atrophy. Ann Neurol 2017; 81: 355-368.
5. Fay AJ, Knox R, Neil EE, et al. Targeted treatments for inherited neuromuscular diseases of childhood. Semin Neurol 2020; 40: 335-341.
6. Kirschner J, Butoianu N, Goemans N, et al. European ad-hoc consensus statement on gene replacement therapy for spinal muscular atrophy. Eur J Paediatr Neurol 2020; 28: 38-43.
7. Leon-Astudillo C, Byrne BJ, Salloum RG. Addressing the implementation gap in advanced therapeutics for spinal muscular atrophy in the era of newborn screening programs. Front Neurol 2022; 13: 1064194.
8. Patel A, Toro W, Bourke S, et al. Treatment preferences in spinal muscular atrophy: a swing weighting study for caregivers of patients with SMA types 1 and 2. PLoS One 2024; 19: e0309666.
9. Ricci F, Vacchetti M, Brusa C, et al. New pharmacotherapies for genetic neuromuscular disorders: opportunities and challenges. Expert Rev Clin Pharmacol 2019; 12: 757-770.
10. Ryan MM. Gene therapy for neuromuscular disorders: prospects and ethics. Arch Dis Child 2022; 107: 421-426.
11. Schorling DC, Pechmann A, Kirschner J. Advances in treatment of spinal muscular atrophy – new phenotypes, new challenges, new implications for care. J Neuromuscul Dis 2020; 7: 1-13.

*Nine articles describing the landscape of advanced therapies and experimental neurotherapeutics in paediatric epilepsies, movement disorders, neuroimmune and neurodevelopmental conditions.*

1. Aranca TV, Jones TM, Shaw JD, et al. Emerging therapies in Friedreich's ataxia. Neurodegener Dis Manag 2016; 6: 49-65.
2. Berg AT, Gaebler-Spira D, Wilkening G, et al. Nonseizure consequences of Dravet syndrome, KCNQ2-DEE, KCNB1-DEE, Lennox-Gastaut syndrome, ESES: a functional framework. Epilepsy Behav 2020; 111: 107287.
3. Byrne S, Enright N, Delanty N. Precision therapy in the genetic epilepsies of childhood. Dev Med Child Neurol 2021; 63: 1276-1282.
4. Goldstein HE, Poliakov A, Shaw D, et al. Precision medicine in pediatric temporal epilepsy surgery: optimization of outcomes through functional MRI memory tasks and tailored surgeries. J Neurosurg Pediatr 2022; 30: 272-283.
5. Paton MCB, Finch-Edmondson M, Fahey MC, et al. Fifteen years of human research using stem cells for cerebral palsy: a review of the research landscape. J Paediatr Child Health 2021; 57: 295-296.
6. Soo AKS, Ferrini A, Kurian MA. Precision medicine for genetic childhood movement disorders. Dev Med Child Neurol 2021; 63: 925-933.
7. Vogt L, Quiroz V, Ebrahimi-Fakhari D. Emerging therapies for childhood-onset movement disorders. Curr Opin Pediatr 2024; 36: 331-341.
8. Wells E, Hacohen Y, Waldman A, et al. Neuroimmune disorders of the central nervous system in children in the molecular era. Nat Rev Neurol2018; 14: 433-445.
9. Zubair U, Agianda HAP, Yang K, et al. DBSMatchMaker: connecting clinicians globally for deep brain stimulation in rare diseases. Mov Disord 2025; 40: 765-767.

## 1.4 – Topic generation

Following the initial scoping review, the steering committee (NL, MF, DSK, CM) convened to distil the broad evidence base into a manageable and focused framework for the Delphi process. Questions to inform and guide the first workshop were generate from iterative discussion between steering committee members and the evidence base. Questions were prioritised according to the impact on safety and equity of therapeutic delivery, information and support needed for families and standardisation and streamlining of evidence generation upon implementation. These questions were grouped into eight core topics to serve as the foundation for the first workshop.

**Supplementary Table 1. Conceptualisation of eight core topics and guiding questions in the first workshop**

| Disease suitability | What do we need to know about individual disease mechanisms and characteristics to prepare and inform for the potential development of target treatments? |
| --- | --- |
| Patient suitability | How should individual clinical factors be assessed to guide patient selection for advanced therapies?  What social and psychological factors must we consider to support treatment adherence?  What strategies can ensure equitable access to advanced therapies across regional, rural and metropolitan areas and among diverse socioeconomic groups? |
| Consent | How to ensure realistic risk-benefit perceptions and clear expectations when counselling families?  What resources are necessary to support a sustainable, thorough consent and communications processes across diverse diseases and families? |
| Study design | Do all stakeholders understand the available access pathways for advanced therapies?  How can clinical trials in small, rare disease populations generate meaningful and generalisable insights?  Are parental and family perspectives being meaningfully integrated into study design and decision-making processes?  How is new data on safety and effectiveness shared publicly, transparently and without delay? |
| Study implementation | How can clinical care be effectively integrated with and inform the Advanced Therapeutics research pipeline?  What strategies can managed the new therapeutic emergencies and increased resource demands that implementation of advanced therapies may create? |
| Infrastructure | Do we have sustainable clinical infrastructure with necessary expertise to assess, treat and monitor patients receiving advanced therapies?  What systems ensure rigorous follow up, safety reporting and ongoing training?  Are short- and long-term patient outcomes (including through transition) able to be captured? |
| Data collection | What are important outcome measures and endpoints particular to advanced therapeutics and experimental neurotherapeutics?  How can technologies like artificial intelligence and machine learning manage and analyse the huge datasets generated by precision medicine development and implementation? |
| Ethics | What are the overarching clinical and research ethics frameworks that relate to the implementation of advanced therapies in paediatric neurology? |

#

## Section 2 – Workshops and Questionnaires

## 2.1 - Workshop 1: Expert panel consultation and thematic development

The first workshop was undertaken on the 24^th^ of November 2023 via the Zoom virtual platform. The expert panel were initial briefed by the moderator (NL) to explain the scope and key definitions. To ensure consistency across the breakout session, the key topic of consent was facilitated as a whole group discussion. Following this, participants were allocated into smaller breakout rooms according to skills and expertise mix to address the remaining topics.

The workshop utilised a qualitative descriptive approach to capture the panel’s expertise. Expert comments were recorded and transcribed then transcripts were screened for key phrases and recurring ideas. These phrases were then categorised into overarching thematic domains.

**Supplementary table 2. Thematic framework and indicative keywords extracted from Workshop 1.**

| **Theme** | **Topics** | **Key words and phrases** |
| --- | --- | --- |
| Access and responsibilities for trials and treatment | Disease suitability, Patient suitability, Ethics | Equity of access; Transparency; Consumer consultation for underrepresented groups; Peer review group; Knowledge exchange; Preimplementation goal setting; Online meetings; Manage conflicts of interest; Recognize blind spots; Address opportunity costs; Family driven processes lack equity; Wider ethics review lens. |
| Communication and engagement with families | Consent, Ethics | Robust patient education; Known knowns (documented side effects, monitoring, follow ups); Known unknowns (unexpected findings, clinical benefits); Documented start and stop criteria; Collaboration with community service and advocacy groups; Potential for bias; Clinician/researcher values may influence parental decision; Risk appetite of families varies; Managing expectations; Psychological support may not be feasible but remains important; Follow up point of contact separate to specialist; Potential need for externality of care or information coordination. |
| Trial and treatment eligibility and patient selection | Disease suitability, Patient suitability, Study implementation | Different risk profiles; Disease severity; Treatment type (repurposed medications vs gene therapy); Route of administration; Level of experience of the site; Reliability of family in follow-up; Stratifying risk is a difficult burden; Conflict of interest; Academic improvement vs clinical recommendation; Prolonging life vs improving quality of life; Timing is critical for optimum therapeutic windows. |
| Evaluating the effects of trials or treatment and implementing a treatment plan | Infrastructure, Data collection, Study design, Ethics | Avoid reinventing the wheel; Avoid repeat mistakes; MTOP applications carry administrative and logistical load; Flexibility of process; Start and stop criteria are dynamic and can be revisited; Therapeutic burden; Disease specific measures; Objective and measurable outcomes; Community engagement; Codesign; Collaboration with families to define outcome measures; Data sharing strategy; Risks of data sharing; Registry development; Feasibility of independent data and safety review. |

## 2.2 - Questionnaire 1

The findings from the literature review and the thematic outputs from Workshop 1 were synthesised into specific, actionable statements to be included in Questionnaire 1. The questionnaire was administered via the REDCap platform and utilised a 5-point Likert scale (Strongly Agree, Agree, Neither Agree nor Disagree, Disagree or Strongly Disagree). An open-ended text box was provided for each item for qualitative feedback. Participants could leave a question blank and indicate a reason i.e. beyond scope of experience. Consensus was predefined as the following:

Consensus to Include ≥ 75% of participants selected "Agree" or "Strongly Agree."

Consensus to Exclude ≥ 75% of participants selected "Disagree" or "Strongly Disagree."

The link to Questionnaire 1 was distributed via email on July 8^th^ 2024.

**Supplementary Table 3. Results that achieved consensus following Q1 across four key domains.**

**Domain 1 Consensus based practice recommendations on the access and responsibilities for trials and treatment**

| Recommendations | Agree % |
| --- | --- |
| 1.1 A collaborative paediatric clinical trials alliance by which the experience and expertise of those studying and utilising advanced and experimental therapeutics can be shared efficiently and transparently should be a priority in local and national policy. | 100 |
| 1.2 A peer review advisory group involving clinicians with expertise in advanced and experimental neurotherapeutics would be valuable as an adjunct to established organisational pathways for clinicians to evaluate best practice, support decision making and share knowledge and experience. | 100 |
| 1.3 The decision to pursue access to unapproved advanced or experimental neurotherapeutics should occur according to established organisational pathways with consideration of the following: |  |
| Appropriate access pathway for an experimental neurotherapeutic | 94 |
| The risks, benefits and uncertainties of the experimental neurotherapeutic based on preclinical and clinical data | 94 |
| The ability of the health system to accept responsibility within a local policy framework. | 94 |
| The capacity of the clinical and health service to implement the proposed treatment and post-treatment activities. | 94 |
| Ethical and social issues, for example funding, equality, and sustainability. | 88 |
| 1.5 Core characteristics of a research network/infrastructure should include: |  |
| Recognition of the impact of their child's condition on the mental health and wellbeing of the family. Proactive access to mental health resources should be discussed and facilitated. | 100 |
| Appropriate authorisations and approvals pathways for accessing and administering the therapy. | 94 |
| The institution and clinician willingness to accept formal responsibility within a local policy framework. | 88 |
| Appropriate resources including personnel, supply, storage and administration facilities are available to enable the delivery of the experimental neurotherapeutic or advanced therapy. | 100 |
| Appropriate expertise (including education and training) developed within the healthcare system to conduct all pre and post treatment activities and administer the treatment safely. | 100 |
| Ongoing comprehensive care for the child from a multidisciplinary team is provided. | 94 |
| Formulation of a plan to facilitate ongoing access for effective neurotherapeutics within the resources of the healthcare system based on principles of equity, scalability and sustainability. | 88 |
| 1.6 In contexts where parents/caregivers are involved in the funding and/or development of therapies, an independent, scientific and ethics evaluation needs to be performed by local organisations with the following considerations: |  |
| Potential conflicts of interest including partnerships with not-for-profit organisations and philanthropic foundations. | 94 |
| Formulation of a plan to incorporate equitable access for other suitable patients and families. | 100 |
| Privacy risks in the case of crowdfunding or compelling cases receiving media attention. | 94 |
| 1.7 Health services should aim to provide transparent and equitable opportunities of access to experimental therapeutics and advanced therapies for children with neurological conditions. | 100 |
| 1.7.1 Where there is competing space on a clinical trial or managed access program and after review of capacity and anticipated clinical benefit, the process of allocation to receive treatment should be transparent and communicated to families. | 100 |
| 1.7.2 Health services should formulate and implement a dissemination plan so that treatment opportunities are shared across the population. | 100 |
| 1.7.3 The dissemination plan should have specific strategies to target underrepresented groups, including but not limited to families with low health literacy, socioeconomic disadvantage, culturally and linguistically diverse populations, Aboriginal and Torres Strait Islander and rural/regional children and their families. | 94 |
| 1.7.4 Health services should collaborate with community services and patient advocacy groups to facilitate dissemination of treatment opportunities to children and families with neurological conditions. | 94 |

**Domain 2 Consensus based practice recommendations on communication and engagement with families**

| Recommendations | Agree % |
| --- | --- |
| 2.1 Once an advanced therapy or experimental neurotherapeutic is identified and approved for administration, the following components should be included in clinician communication and education with the child and family/caregiver and documented: |  |
| The child and family/caregivers should be informed of available treatment options within the Australian healthcare landscape. | 100 |
| For children where treatments are or may be available outside of Australia, the potential benefits, risk (including potential for unregulated treatments and/or unregulated institutions of administration) and uncertainties of these should be discussed. | 88 |
| Families whose primary language is not English should be supported in communication through the use of a professional interpreter. | 100 |
| Families who identify as Aboriginal or Torres Strait Islander descent should have the option of support through the Aboriginal Health Liaison Officer through the communication process. | 94 |
| The child and family/caregiver should be given the option of referral to patient advocacy groups for support during the process of therapeutic decision making. | 100 |
| Where available, children and families/caregivers should be provided with well curated, evidence based written or multimedia resources to support therapeutic decision making. | 100 |
| The need for staged decisions about treatment initiation, continuation and discontinuation. | 100 |
| 2.2 Start and stop criteria should be discussed and established with the child and family/caregivers and documented, ideally early in communications, and before treatment initiation to set expectations. | 100 |
| 2.2.1 The following areas should be included in START criteria discussions: |  |
| Defined treatment objectives | 100 |
| Evaluation of outcomes including frequency, assessment type and duration. | 100 |
| Evaluation of safety signals. | 100 |
| Rationale for treatment that includes scientific justification and plausible efficacy. | 100 |
| 2.2.2 The following areas should be included in STOP criteria discussions: |  |
| Treatment objectives are not met from the perspective of the child and family. | 94 |
| Treatment objectives are not met from the perspective of the managing clinician. | 88 |
| The child’s clinical status deteriorates due to a clinical situation that is difficult to reverse. | 94 |
| The child experiences a severe adverse drug reaction. | 94 |
| The cumulative side effects of the treatment necessitate cessation. | 100 |
| The child and/or family cannot adhere to ongoing treatment and/or clinical surveillance. | 100 |
| If the child deteriorates after cessation of the treatment, then it may be an indication to start again if appropriate. | 88 |
| Children and families/caregivers should be appraised of the potential for cessation or temporary withholding of access to an experimental neurotherapeutic if serious adverse reactions are observed within the international therapeutic landscape. | 100 |
| 2.3 Ideally, as soon as families express interest in advanced therapies, or experimental neurotherapeutics health professionals should: |  |
| Offer connections with appropriate psychological, medical and/or social work support | 100 |
| Offer and facilitate referral to patient advocacy services as appropriate. | 88 |
| Initiate an ongoing dialogue to allow time to make decisions as informed as possible given uncertainties and risks. Ideally this dialogue spans several conversations to optimise information provision. | 100 |
| Describe the process of accessing therapies including the wait for due diligence processes. | 100 |
| Establish expectations of the mode and frequency of communication back and forth. | 100 |
| 2.4 In medical emergencies where delays in treatment can adversely change future outcomes for the child, families/caregivers should be immediately informed of the benefit of early treatment to aide their decision making. | 94 |
| 2.5 Coordination of information provision is recommended and delegated to the most appropriate healthcare professional/s, based on levels of expertise and therapeutic relationship with the family/patient. | 100 |
| The most appropriate healthcare professional(s) for contact regarding experimental trials and treatments should be agreed upon by all and contact details provided. | 92 |
| 2.6 Informed consent needs to involve transparency around the following: |  |
| Anticipated outcomes, including explicitly the potential for a therapy being non efficacious. | 100 |
| The potential to preclude the child in accessing future clinical trials or treatments. | 100 |
| The potential short and long-term side effects and uncertainties of the treatment itself. | 100 |
| The potential side effects or risks of the administration/delivery process. | 100 |
| The nature of treatment including its action. | 100 |
| The treatment related burden including the mode and frequency of administration and pre and post administration surveillance. | 100 |
| The variability in sustained access to experimental neurotherapeutics over the longer term, even if deemed beneficial (relevant within the clinical trial domain). | 100 |
| 2.7 A second clinician or researcher with technical knowledge and understanding of the ethical applications of advanced therapeutics should be offered if there is uncertainty | 100 |

**Domain 3 Consensus based practice recommendations on trial and treatment eligibility and patient selection**

| Recommendations | Agree % |
| --- | --- |
| 3.1 Outside of a clinical trial (which have set inclusion and exclusion criteria), inclusion and exclusion criteria should be reviewed at the point of therapeutic decision making and over time, as the risk-benefit profile may change in the context comorbidities and stage of disease. | 88 |
| 3.2 Inclusion criteria (outside of sponsored clinical trials) consist of the following: |  |
| Informed consent. | 100 |
| Defined and documented expectations about possible benefit. | 94 |
| Defined and documented expectations about possible harms and uncertainties. | 94 |
| The ability of the child and family to continue to receive multidisciplinary care. | 94 |
| Availability of other safe and effective disease modifying treatment is limited. | 100 |
| The treatment has the potential to provide benefit to the child. | 94 |
| 3.2.1 The potential to provide benefit to the child may be informed by patient level characteristics including: |  |
| Associated comorbidities. | 100 |
| Child and family preferences. | 94 |
| Ability of the child and family to adhere to post treatment surveillance. | 88 |
| 3.2.2 The potential to provide benefit to the child may be informed by disease level characteristics including: |  |
| Stage or phase of disease. | 100 |
| Reversibility of end organ damage. | 100 |
| Extent of end organ damage. | 100 |
| 3.2.3 The potential to provide benefit to the child may be informed by scientific justification including: |  |
| Evidence in children with similar disease characteristics and comorbidities. | 94 |
| Evidence of benefit in preclinical models including evidence from animal models, functional tissue and cell-based assays and biomarkers of therapeutic response. | 88 |
| 3.2.4 The benefit of accessing treatments at an advanced stage of a neurodegenerative disease(s) should take into account |  |
| The possibility of symptom stability. | 100 |
| Maintenance of quality of life and adaptive function. | 100 |
| Patient and patient-proxy beliefs pertaining to meaningful treatment outcomes | 92 |
| Conventional endpoints including gains in function, developmental progression and reduction in mortality and comorbidities. | 83 |
| 3.3 Exclusion criteria consist of the following: |  |
| Availability of alternative safe and effective disease modifying treatments. | 92 |
| Not able to safely administer (related to the specific treatment – e.g. anaesthetic risk, bleeding disorder, cardiac, liver, or renal dysfunction, concomitant drug interactions). | 100 |
| Therapeutic burden is too high for the child in the opinion of parents and/or the treating team. | 92 |

**Domain 4 Consensus based practice recommendations on evaluating the effects of trials or treatment and implementing a treatment plan**

| Recommendations | Agree % |
| --- | --- |
| 4.1 For advanced therapies and experimental neurotherapeutics, safety and clinical outcome measures should be defined in advance for each case. | 94 |
| 4.2 It is recommended that outcome measures include a range of meaningful child and family endpoints alongside conventional clinical endpoints. | 100 |
| 4.3 It is recommended that outcome measures include subjective experiences (such as quality of life scales and global impression scores) alongside conventional clinical endpoints. | 94 |
| 4.4 Outcome measures should be appropriate to the disease stage, function, and disease trajectory of children. | 94 |
| 4.5 Outcome measures should be appropriate to the treatment target. | 94 |
| 4.6 Safety assessments should include physical exams and targeted assessments related to known class effects. | 100 |
| 4.7 It is recommended that early engagement with consumers occurs to facilitate co-design of clinical trials and co-development of appropriate endpoints. | 100 |
| 4.8 Potential surrogates (biomarkers) of disease activity and therapeutic effect should be assessed and incorporated into ongoing research. | 94 |
| 4.9 An independent review of data and safety assessments and reports should occur. | 100 |
| 4.10 It is recommended that comparators for trials (outside of sponsored clinical trials) are chosen dependent on the condition and investigational treatment. | 77 |
| 4.11 To allow the best opportunities for clinical trial readiness and real-world evaluation of effects, the following should be fostered, ideally as platforms that can be used across different conditions and therapies: |  |
| Have protocols in place to conduct evaluations prior to administration of treatments E.g., parallel collection of natural history, run in data, use of retrospective data. | 100 |
| Have protocols in place to collect data following initiation of treatment, and potentially long-term. | 100 |
| Have proactive measures established to monitor and strengthen safety. | 100 |
| Have measures for rapid data sharing to accelerate learning. | 100 |
| 4.12 To allow the best opportunities for the clinical development and implementation of new therapies, the following should ideally occur: |  |
| Ongoing education and professional development of health care professionals, ethics and governance staff and health system planners, managers, and administrators. | 100 |
| A local ethics and governance officer specific to advanced therapies. | 83 |
| Community engagement to determine what families and children want and need to know and how best communicate information to improve their understanding and decision making. | 100 |
| Consumer partnerships to co-design health care to meet their needs. | 100 |
| A framework for access and use of new therapies. | 100 |
| Standards for clinicians and centres. | 94 |
| Promotion of multidisciplinary teams and research integrated clinical care models. | 100 |

## 2.3 - Workshop 2: Review of non-consensus items

The second workshop was undertaken on the 28^th^ August 2024 via the Zoom virtual platform. Five discussion points with correlating survey statements were placed on the agenda for discussion.

1. Pathways and personnel for providing information and maintaining communication with parents and families.
2. Start and stop criteria as well as inclusion and exclusion criteria.
3. Assessment of risk compared to potential for benefit.
4. Equity of access.
5. Inclusion of the Medical treatment overseas program.

**Supplementary Table 4. Statements reworded, included or excluded following Q1 with reasons for exclusion and percentage disagreement**

| Statement | Agree % | Outcome |
| --- | --- | --- |
| The decision to pursue access to unapproved advanced or experimental neurotherapeutics should occur according to established organisational pathways with consideration of the following: |  |  |
| Suggestions for innovative trial designs. | 72% | Ambiguous wording. Excluded from Q2. |
| For families wishing to pursue overseas access to clinical trials, new therapies and technologies or unregulated or unproven therapies, the managing healthcare professional should: |  |  |
| Seek advice from local ethics and governance committees and existing trial evaluation assessors as available. | 72% | Out of scope.  Excluded from Q2. |
| Assist in Medical Treatment Overseas Program applications when appropriate. | 72% | Out of scope.  Excluded from Q2. |
| Collaborate with the overseas investigator team to exchange medical information. | 83% | Met consensus but out of scope.  Excluded from Q2. |
| Link families to the Therapeutic Goods Administration advice on buying medicines and medical devices online. | 66% | Out of scope.  Excluded from Q2. |
| Link families to the Smartraveller advice on travelling overseas for a medical procedure. | 61% | Out of scope.  Excluded from Q2. |
| The following areas should be included in STOP criteria discussions: |  |  |
| Treatment objectives are only partly met from the perspective of the child and family. | 66% | Ambiguous.  Excluded from Q2. |
| Treatment objectives are only partly met from the perspective of the managing clinician. | 61% | Ambiguous.  Excluded from Q2. |
| Ideally, as soon as families express interest in advanced therapies, or experimental neurotherapeutics health professionals should: |  |  |
| Facilitate referral to psychological, medical and/or social work support. | 66% | Amended to offer. Included in Q2. |
| A second independent clinician or researcher with technical knowledge and understanding of the ethical applications of advanced therapeutics is recommended in the process of consent. | 72% | Amended to offer  Included in Q2. |
| Exclusion criteria consist of the following: |  |  |
| Availability of alternative safe and effective treatments. | 72% | Amended to safe and effective disease modifying treatments.  Included in Q2. |
| No benefit is anticipated due to patient, disease level factors and scientific justification. | 72% | Considered too rigid.  Excluded from Q2. |

## 2.4 - Questionnaire 2

Following analysis of the first round, Questionnaire 2 was constructed. This version included all statements that had reached consensus, those that required rewording based on panel feedback and new statements derived from the previous round’s quantitative data. The panel were asked to assess the feasibility of implementing each statement. Panel members were asked to evaluate cost, scalability, and required resources including time. The following feasibility scale was provided to panel members at the start of the Questionnaire.

**Very feasible:** Easily implementable in most centres in a short time frame with minimal changes to current resourcing.

**Feasible:** Able to be implemented with some provision of funding and resources.

**Neutral**

**Not feasible:** Current barriers to implementation would require significant change to policy or process that may not be achievable.

**Very not feasible:** Extremely difficult and complex to implement; not realistic or possible.

The link to Questionnaire 2 was distributed via email on November 14^th^ 2024.
